# Preventive Effect of Quercetin in a Triple Transgenic Alzheimer’s Disease Mice Model

**DOI:** 10.3390/molecules24122287

**Published:** 2019-06-20

**Authors:** Pérez-Corredor Paula, Sabogal-Guáqueta Angelica Maria, Carrillo-Hormaza Luis, Cardona-Gómez Gloria Patricia

**Affiliations:** 1 Neuroscience Group of Antioquia, Cellular and Molecular Neurobiology Area, Faculty of Medicine, SIU, University of Antioquia, Calle 70 # 52-21, Medellín, Colombia; paula.perezc@udea.edu.co (P.-C.P.); angelica.sabogal@udea.edu.co (S.-G.A.M.); 2 Group de Investigación en Sustancias Bioactivas, Facultad de Ciencias Farmacéuticas y Alimentarias, Universidad de Antioquía, Cra. 53 #6739, Medellín, Colombia; Lcarrillo89@gmail.com

**Keywords:** Alzheimer’s disease, prevention, quercetin

## Abstract

Alzheimer’s disease (AD) is the most common type of dementia and is the leading cause of disability in elderly people worldwide. Current pharmacological therapies do not cure the disease, and for this reason, some pharmacotherapy studies have investigated preventive treatments focused on modifiable nutritional factors such as diet. Quercetin (Qc) is a flavonoid found in fruits and vegetables that has several biological properties. In this study, we evaluated the effect of chronic oral quercetin administration (100 mg/kg) on neurodegeneration markers and cognitive and emotional deficits in a triple transgenic Alzheimer’s disease (3xTg-AD) mouse model using histological and behavioral analyses. Our results suggest that long-term (12 months) oral preventive treatment with quercetin has significant effects on β-amyloidosis reduction and tends to decrease tauopathy in the hippocampus and amygdala. These decreases positively affected the cognitive functional recovery (without modifying the emotional skills) of 3xTg-AD mice. These findings suggest that preventive and chronic administration of Qc might help to delay the development of histopathological hallmarks and cognitive function deficits in AD.

## 1. Introduction

The life expectancy has been increasing worldwide, and it is estimated that by 2030 there will be almost 1.4 billion people over the age of 60 [1]. Due to the increased population of this age group, Alzheimer’s disease (AD) and other types of dementias have become more frequent because their main risk factor is increasing age. According to the World Health Organization, there are approximately 47.5 million people suffering from dementia, and there are 9.9 million new cases each year [1]. Dementia of Alzheimer’s type is a progressive neurodegenerative disorder characterized by a decreased cognitive function that usually begins with memory impairment. It is characterized by the accumulation of beta amyloid (βA) and the hyperphosphorylation of tau, resulting in the formation of amyloid plaques and neurofibrillary tangles, respectively [2]. These neuropathological markers induce the loss of synaptic connections in specific regions of the brain, neuroinflammation and a high rate of oxidative stress that triggers protein oxidation, lipid peroxidation, and DNA oxidation, thereby activating neuronal apoptosis [3,4,5,6]. Despite an understanding of these cell death mechanisms and several years of invested research, no drug has been successfully approved for the treatment of AD [7]. In addition, recent research suggests that changes in people’s lifestyles, such as their diet, could also affect the incidence of dementia [8,9,10].

The majority of potential AD therapies that have been evaluated in animal models do not work or have limited action when they are transferred to humans due to several types of comorbidities associated with the cause and progression of AD [11]. Quercetin (Qc) is a molecule with a broad range of biological properties impacting several diseases. It has been attributed to have anticancer, anti-inflammatory, anti-atherosclerotic, antithrombotic, and antihypertensive activities, and it modulates the resistance to different drug [12,13,14,15,16]. Flavonoids are powerful eliminators of reactive oxygen and nitrogen species, which are associated with oxidative stress [16]. Although the mechanisms of neuroprotection by Qc are not completely elucidated, its neural effect has been demonstrated in in vitro and in vivo models [17,18]. For example, in our previous study, we demonstrated that Qc administered for 3 months by IP injection decreases extracellular β-amyloidosis and tauopathy and protects the cognitive and emotional function in old triple-transgenic model mice with Alzheimer’s disease (3xTg-AD) [19]. However, it has not been evaluated whether long-term oral treatment with quercetin might prevent cognitive impairments prior to the development of histopathological hallmarks. The main aim of the current study was to investigate this question to validate the translational potential of the therapy to humans. 

## 2. Results

### 2.1. Preventive Quercetin Treatment Decreases Neurodegeneration Markers in 3xTg-AD Mice

**Figure 1:**
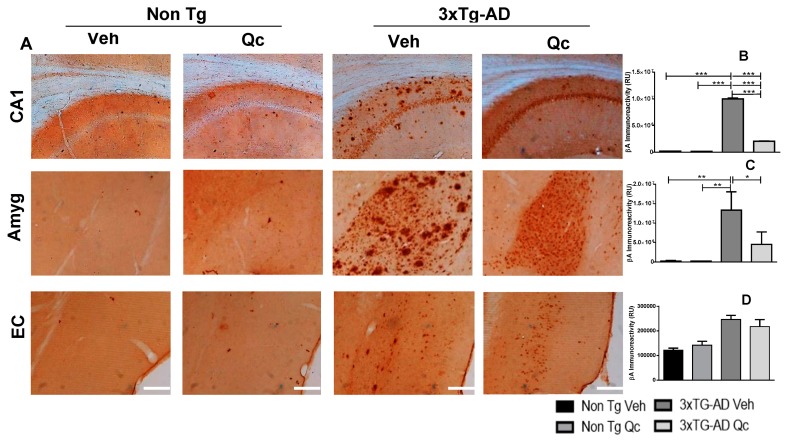
Quercetin oral administration effect on the prevention of β-amyloid plaques in triple transgenic Alzheimer’s disease (3xTg-AD) mice. (**A**) Representative images of βA immunoreactivity in the CA1 region of the hippocampus, the amygdala, and the entorhinal cortex of 19-month-old 3xTg-AD and non-Tg mice treated with vehicle and quercetin. Magnification: 4x. Scale bar: 50 μm. The values in the immunoreactivity graphs are expressed in relative densitometric units (RU) in CA1 of the hippocampus (**B**) Amyg: amygdala (**C**) and EC: entorhinal cortex (**D**). Veh: vehicle (DMSO 0.5%). n = 3. * *p*: < 0.05, **p: < 0.001, ***p: < 0.0001.

**Figure 2:**
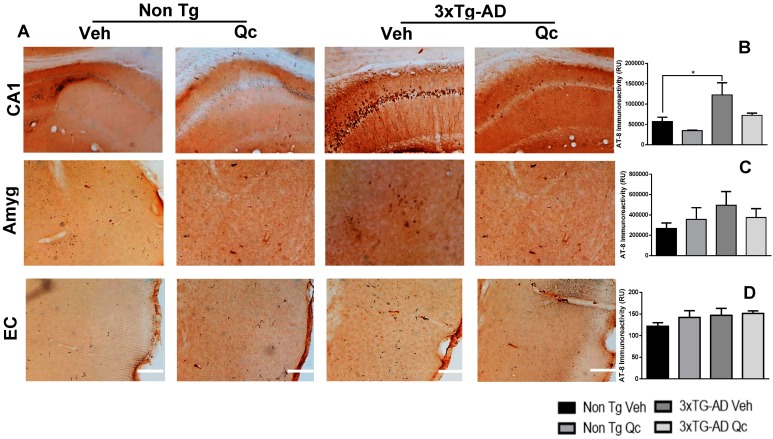
Quercetin oral administration effect on the prevention of hyperphosphorylation of tau. Representative images of the AT-8 immunoreactivity in the CA1 region of hippocampus, the amygdala and the entorhinal cortex of 3xTg-AD mice and non-Tg 19-month-old mice treated with vehicle and quercetin (**A**). Magnification: 10x. Scale bar: 50 μm. The values in the immunoreactivity graphs are expressed in relative densitometric units (RU) in the CA1 of the hippocampus (**B**) Amyg: amygdala (**C**) and EC: entorhinal cortex (**D**). Veh: Vehicle (DMSO 0.5%), Qc: quercetin. n = 3. * *p*: < 0.05.

Because tau and β-amyloid are the main neurodegeneration hallmarks in AD and develop progressively in humans and in the 3xTg-AD mouse model, we analyzed the preventive effect of quercetin in the apparition of these markers after 1 year of oral administration. When the Qc treatment was finished, brain sections were selected in the Bregma −2.46 mm to analyze these neurodegeneration markers by immunohistochemistry in the hippocampus, amygdala, and entorhinal cortex (Figure 1 and Figure 2). The data showed that β-amyloid immunoreactivity increased significantly in CA1 of the hippocampus and in the amygdala of 3xTg-AD mice, with a slight tendency at the entorhinal cortex (EC) in comparison with non-Tg mice, with and without Qc. Interestingly, oral treatment with the flavonoid strongly prevented β-amyloid aggregation in the CA1 and amygdala, without changes in the EC (Figure 1). Additionally, Qc treatment did not generate changes in the weights of the mice (data not shown). 

Complementing the above findings, the AT-8 antibody showed a significant increase of hyperphosphorylated tau in the CA1 region of the hippocampus and in the amygdala of 3xTg-AD Qc mice. These effects were partially prevented by Qc, showing a similar aspect and densitometry as the control groups. The EC did not present AT-8 changes in the groups (Figure 2).

### 2.2. Preventive Quercetin Treatment Protects Cognitive Function in 3xTg-AD Mice

**Figure 3:**
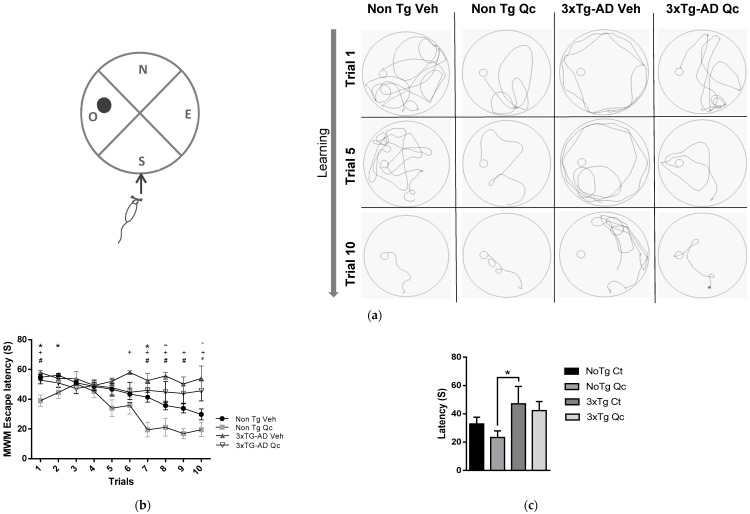
Effect of quercetin oral administration on the spatial learning test in 3xTg-AD mice. (**A**) Representative images of the trajectory in the MWM in one of the four positions in trials 1, 5, and 10. (**B**) Exhaust latency in the spatial learning test. (**C**) Latency of passing through the platform in the learning retention test. The time is expressed in seconds. Data are represented as the mean ± SEM. n = 8–19. * *p*: < 0.05; ** *p*: < 0.01; *** *p*: 0.001. * Differences between non-Tg Veh and non-Tg Qc; - Differences between non-Tg Veh and 3xTG-AD Veh; + Differences between non-Tg Qc and 3xTG-AD Veh; # Differences between non-Tg Qc and 3xTG-AD Qc.

It has been described that 3xTg-AD animals have a higher escape latency in the Morris water maze (MWM) [19,20], which was supported by a longer trajectory of the 3xTg-AD mice in the pool in trials one, five, and ten (Figure 3A), and a slight but significantly reduced latency is observed when comparing Qc-treated 3xTg-AD mice with untreated animals (Figure 3B). These learning processes may be related to the decreased plaques of βA and hyperphosphorylated tau in the CA1 area of the hippocampus. In addition, Qc alone improved the learning task performance of the non-Tg mice compared with the untreated control group (Figure 3B), mainly in the last four trials. However, the memory tasks did not show a complete recovery after the treatment in the AD mice, only showing a weak tendency to reduce the escape latency in the treated non-Tg and 3xTg-AD groups with respect to the untreated groups (Figure 3C). 

### 2.3. Quercetin Tends to Improve Active Behaviors of 3xTG-AD Mice

**Figure 4:**
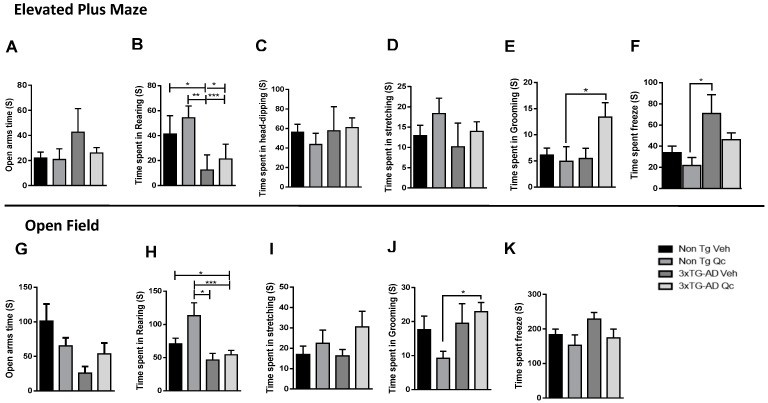
Effect of quercetin oral administration on the emotional behavior of 3xTg-AD mice. Elevated plus maze: Time spent on the open arms (**A**). Time spent rearing (**B**), head-dipping (**C**), stretching (**D**), grooming (**E**), and freezing (**F**). Open field: Time in the center (**G**). Time spent on rearing (**H**), stretching (**I**), grooming (**J**), and freezing (**K**). The measurement of time is expressed in seconds. The data are represented as the mean ± SEM. n = 4–19. * *p*: < 0.05; ** *p*: < 0.01; *** *p* < 0.001.

The emotional behavior was evaluated to analyze the effect of Qc on the amount of time spent in typical behaviors, such as stretching, grooming, rearing, and head-dipping, in the 3xTg-AD mice by the elevated plus maze (EPM) and open-field (OP) tests. We observed that the frequency and time were very similar, so only the time spent in each behavior is presented. In the EPM test, the 3xTG-AD Veh animals tended to spend more time in the open arm (Figure 4A), and the 3xTG-AD Qc mice had values very similar to the non-Tg Veh and non-Tg Qc mice, without changes in risk assessment behaviors such as rearing, stretching, and head-dipping (Figure 4B–D). However, the 3xTG-AD Qc animals spent more time grooming than the 3xTG-AD Veh group, which had similar times to the non-Tg Veh mice in this behavior (Figure 4E). Additionally, the 3xTG-AD Veh mice spent more time freezing, which was reduced by Qc treatment (Figure 4F), similar to the results obtained for the control groups. These data were supported by more time spent in grooming (Figure 4J), and a tendency for increased rearing (Figure 4I) and decreased freezing (Figure 4K) by the 3xTg-AD Qc mice in the open field test, without significant changes in the other evaluated behaviors (Figure 4 G–I).

## 3. Discussion

In this study, for the first time, we propose a primary prevention strategy for reducing the neurodegeneration hallmarks and cognitive impairment of aged 3xTg-AD mice through the one-year oral administration of quercetin, starting before β-amyloid spreading in the hippocampus and without the presence of tauopathy at 6 months [21]. This finding implies that the molecule prevents amyloid aggregation and PHF formation and/or its accumulation over the studied time period. The results support that the use of this early therapy for AD could provide promising results [22], not only through reversing the markers in late-stage AD in the 3xTgAD mice model (as we previously observed when applying intraperitoneal administration [19]), but also by maintaining its protective properties, as has been suggested by Dajas, 2015 [23]. Moreover, in vitro studies have demonstrated that quercetin protects neurons against the cytotoxicity, protein oxidation, lipid peroxidation, and apoptosis caused by βA and regulates the activity of antioxidant genes [24,25], supporting its action as an antioxidant, anti-inflammatory, anticancer, anti-stress, and antidiabetic compound [16,23,26,27].

It is probable that these beneficial effects are reflected in the improved learning performance of the non-Tg mice treated with quercetin compared to that achieved in the 3xTgAD group. Although we detected better learning in the AD mice, there were no changes in the retention test, which evaluates memory, maybe because consolidation is dependent on the interactions of the hippocampus with other areas such as the amygdala and entorhinal cortex [28], which were less able to be recovered by the treatment.

The amygdala is part of the limbic system and is involved in attention, perception, emotional memory, declarative memory, and explicit memory [29]; although there was a significant reduction of β-amyloid in the amygdala, which could partially explain the weak effect of Qc on the emotional tasks, only a tendency toward more active behaviors (i.e., less freezing, more rearing, and more grooming) similar to that of the control groups was observed, suggesting better neuronal connectivity in the 3xTg-AD mice. There were no AT-8 immunoreactivity changes in the amygdala and EC in mice, which is in agreement with previous studies [19,30]. Therefore, the anxiolytic role of quercetin is not conclusive, in contrast to other studies [19,27,31,32]. The findings of the previous studies probably resulted from decreased targeting of the molecule in the EC in the animal model, thus showing the main action in the CA1 hippocampal region. 

The neuroprotective mechanisms of quercetin are controversial because the in vitro results are very clear but the in vivo results are not; in some investigations, quercetin causes neuroprotection, but others do not show neuroprotection even after long-term oral administration [23]. One of the variables related to this issue could be the age of the animals upon quercetin administration. As in humans, when AD is in an advanced stage, it is very difficult to reverse the neurodegeneration process. It is highly probable that Qc treatment does work in humans, considering that this compound has similar bioavailability properties in rats and humans and it has no differences by sex [33]. However, previous studies report that the natural powder extract of foods enriched in Qc have better availability than dihydrate powder [33], and also work better in the food matrix [34]. In addition, chronic administration and daily doses should be specifically analyzed because some side effects have been reported, showing predisposition to primary renal damage and breast cancer susceptibility [35]. Therefore, studies controlling those variables, specifying doses per type of comorbidity, and clinical trials including middle cognitive impairment patients should be developed. 

Nevertheless, with our present study, we are close to discovering a preventive intervention that could be successful in decreasing the probability of βA or tau accumulation in the brain, and in this way, prevent the toxic environment that initiates the neurodegeneration cascade. Further, AD is a multifactorial disease where the toxic context could be the result of different insults to the body, and quercetin has been shown to have beneficial effects in many diseases, such as hypertension, diabetes, and atherosclerosis, as well as against oxidative stress and inflammation [12,13,14,15,16]. Therefore, this molecule is a good candidate for the prevention of Alzheimer’s disease, but the availability of this compound must be improved to achieve more general and conclusive results. 

## 4. Materials and Methods 

### 4.1. Animals

Homozygous triple-transgenic 3xTg-AD mice (PS1_M146V_ knock-in, APP_swe_, tau_p301L_) and PS1_M146V_ knock-in mice (named non-Tg) from the in-house colony at the University of Antioquia that were maintained at the SIU (Sede de Investigación Universitaria) specific pathogen-free vivarium in Medellin, Colombia, were used at 6 months old [21]. The mice were maintained on a 12:12 h dark:light cycle and received food and water ad libitum. The animals were handled according to Colombian standards (law 84/1989 and resolution 8430/1993) and guidelines. Special care was taken to minimize animal suffering and to reduce the number of animals used. The animals were weighed at 6, 10, 13, and 16 months, and a genetic control was applied to all procedures that involved the 3xTG-AD mice. The genotype was verified using liver and tail tissue, following the Antioquia Neuroscience Group’s genetic control protocol for 3xTG-AD mice. 

### 4.2. Quercetin Administration

Quercetin was obtained from a commercial source (Cayman Chemical, Cat: 10005169) and dissolved at a concentration of 100 mg/kg in saline solution containing 0.5% dimethyl sulfoxide (DMSO). The administration began when the animals were 6 months old and took place by gavage every 48 h for 1 year.

### 4.3. Elevated Plus Maze

To evaluate anxiolytic activity, the animals were exposed to the elevated plus maze (EPM). The EPM was composed of white Plexiglas and was illuminated at approximately 30–40 lux. The apparatus consisted of two open arms (30 × 5 × 0.25 cm) and two closed arms (30 × 5 × 15 cm) extending from a common central platform (5 × 5 cm). The entire apparatus was elevated on a single central support to a height of 60 cm above the floor. Each mouse was placed in the middle section facing an open arm and was allowed to explore the maze for a single 5 min session during which the experimenter was out of view. After each trial, the maze floor was wiped clean with 10% alcohol.

The following parameters were recorded: frequency of open and closed arm entries (arm entry was defined as all four paws in the arm), total arm entries, and the amount of time spent by the animals in the open and closed sections of the maze. These data were used to calculate the % open or closed arm entries (e.g., open entries/total entries × 100) and the % time spent in the open or closed arms (e.g., open time/300 × 100). The frequency and duration of standard behaviors were measured, including rearing (all rearing occurred against the walls of the enclosed arms), as well as discrete behaviors such as head dipping (exploratory movement of the head/shoulders over the side of the maze), stretched attentive postures (stretching, an exploratory posture in which the mouse stretches forward and retracts to the original position without traveling forward), grooming (a species-typical sequence beginning with the snout, progressing to the ears and ending by grooming the entire body), and freezing (remaining motionless). Each experiment was videotaped using a high-resolution video camera. The data were collected and analyzed using X-Plo-Rat 2005 software.

### 4.4. Morris Water Maze

A white plastic tank 1 m in diameter and 30 cm in height was filled with water (22 ± 2 °C) to a depth of 20 cm. A platform (7 cm diameter) was placed 1.5 cm below the surface of the water during the spatial learning task and 1.5 cm above the surface of the water during the visible session. Extra visual cues around the room remained in a fixed position throughout the experiment. Ten sessions or trials were performed, two complete sessions per day, for five days (Figure 1). Each session consisted of four successive subtrials (30 s intertrial interval), and each subtrial began with the mouse placed pseudorandomly in one of four starting locations. The animals had been trained to stay on the platform for 30 s prior to the initial trial. The latency to reach the platform was evaluated using a visible platform to control for any difference in visual-motor abilities or motivation between the experimental groups. If a mouse did not locate the platform after a maximum of 60 s, it was gently guided to the platform. The animals were then given 48 h of retention time, followed by a probe trial of spatial reference memory in which the animals were placed in the tank without the platform for 60 s (Figure 1). The latency to reach the exact former platform location and the number of crossings of the platform target quadrant were recorded during the probe trial. An automated system (Viewpoint, Lyon, France) recorded the behavior of the animals.

### 4.5. Immunohistochemistry

The tissue sections were initially treated with methanol (50% *v*/*v*) and hydrogen peroxide (30% *v*/*v*) in 0.1 M PBS (pH 7.4) for 20 min to inhibit endogenous peroxidase activity. Three washes with 0.1 M PBS were then performed, and the nonspecific binding sites were blocked for 1 h using a preincubation solution consisting of 0.1 M PBS containing BSA (1%) and Triton X-100 (0.3% *v*/*v*). Next, the sections were incubated overnight at 4 °C in a primary antibody that was diluted in incubation solution (0.3% BSA and Triton X-100 (0.3% *v*/*v*) in 0.1 M PBS). Anti-βA (monoclonal 1–16 (6E10), #SIG-39320, Covance, 1:500) and anti-phospho-PHF-tau (pSer202/Thr205 (AT8), #MN1020, Thermo Scientific, 1:500) antibodies were used. The next day, the sections were washed three times in 0.1 M PBS for 5 min each and then incubated for 1 h at room temperature with the secondary antibody (1:250 dilution, biotin-conjugated goat anti-rabbit IgG (H + L), #31822; or biotin-conjugated goat anti-mouse IgG (H + L), #31800, Pierce), depending on the host species from which the primary antibody was prepared.

After three washes with 0.1 M PBS, the tissues were incubated in avidin-biotin complex (1:250 reagents A and B, ABC Standard Peroxidase Staining Kit, #32020, Pierce) for 1 h. After the complex was removed, three additional washes were performed, and diaminobenzidine (DAB) was used to develop the reaction. Subsequently, the sections were dehydrated using an alcohol series, cleared with xylene, and sealed using Consult-mount. The quantification of immunoreactivity in the examined areas was determined using a 10× or 40× objective and was analyzed using Fiji ImageJ 1.45 software (NIH; Madison, WI, USA). The tissues incubated in the absence of a primary antibody did not display immunoreactivity. The regions including the CA1 (hippocampus), the entorhinal cortex (EC), and the amygdala were evaluated at Bregma −2.46 mm (Paxinos and Franklin, 2004).

### 4.6. Experimental Design

**Figure 5:**
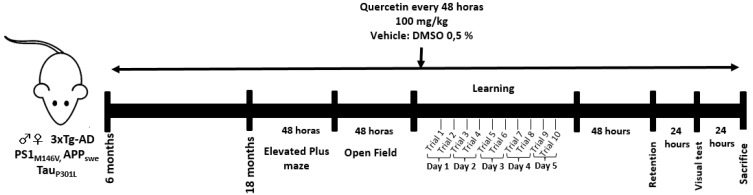
Experimental design of preventive treatment with quercetin.

Quercetin (100 mg/kg) and 0.5% DMSO were administered orally every 48 h for one year to 6-month-old knock-in PS1 and 3xTG-AD (knock-in PS1, APP and tau) mice. When the mice were 18 months old, they were tested for EPM and OP (over the course of 4 days), and MWM (learning, retention and visible test) was performed over the course of 9 days. The animals were then sacrificed for histological analysis (Figure 5).

### 4.7. Statistics

A total of 3 mice were used for each immunohistochemistry assessment, and 4–19 mice were used for each behavioral assay. We applied the Shapiro–Wilk normality test to the data. The parametric data were evaluated via analysis of variance (ANOVA) to compare the 4 groups, followed by Tukey’s test for post hoc multiple comparison between-group analyses. The nonparametric data were evaluated using the Kruskal–Wallis test. The escape latency in the training test was determined via two-way ANOVA, followed by Tukey’s post hoc test for multiple comparisons. The statistical analyses were performed using GraphPad Prism software (version 6.0), and the results were significant at *p* ≤ 0.05. The values are expressed as the means ± SEM.

## 5. Conclusions

Our study proposes that quercetin could be a promising primary preventive strategy for Alzheimer’s disease. However, it is necessary to improve the bioavailability of quercetin by oral administration and to define a dose for long-term administration in humans within the perspective of a translational therapy.
